# Web-Based Cognitive Training: Patient Adherence and Intensity of Treatment in an Outpatient Memory Clinic

**DOI:** 10.2196/jmir.3377

**Published:** 2014-05-07

**Authors:** Vítor Tedim Cruz, Joana Pais, Ivânia Alves, Luís Ruano, Cátia Mateus, Rui Barreto, Virgílio Bento, Márcio Colunas, Nelson Rocha, Paula Coutinho

**Affiliations:** ^1^Hospital São SebastiãoNeurology DepartmentCentro Hospitalar de Entre o Douro e VougaSanta Maria da FeiraPortugal; ^2^Clinical Research OfficeHealth Sciences DepartmentUniversity of AveiroAveiroPortugal; ^3^University Institute of MaiaMaiaPortugal; ^4^UnIGENeInstituto de Biologia Molecular e CelularUniversity of PortoPortoPortugal

**Keywords:** cognitive training, neurorehabilitation, Web-based training, eHealth systems, training intensity, adherence, memory clinic

## Abstract

**Background:**

Cognitive training has been playing an increasing role in the treatment of patients with cognitive deficits. This type of intervention, namely its intensity, can be optimized by incorporating information technology-based systems.

**Objective:**

The intent of the study was to determine the treatment intensity and patient adherence to home-based cognitive training strategies (Web-based cognitive training).

**Methods:**

A cohort of 45 patients with neurologic and psychiatric diseases attending an outpatient memory clinic (average age 50.7 years, SD 17.0; average education 7.8 years, SD 4.9) was followed over 18 months. Participants were challenged to use a Web-based cognitive training system, “COGWEB”, on a daily basis, and fulfilled at least four weeks of training supervised remotely. Additionally, 11 patients attended face-to-face sessions.

**Results:**

The average duration of continuous cognitive training was 18.8 weeks (SD 18.9). Each patient performed on average 363.5 minutes/week (SD 136.6). At 6-month follow-up, 82.8% complied with their treatment plan. The average proportion of complete weeks was 0.75 (SD 0.22). Patients with dementia trained more intensively (444.6 minutes/week), followed by patients with static brain lesion (414.5 minutes/week; *P*=.01). The group that held face-to-face sessions performed more training overall (481.4 vs 366.9 minutes/week), achieving a stronger expression and statistical significance in the last week of training (652.6 versus 354.9 minutes/week, *P*=.027).

**Conclusions:**

Overall, the weekly training intensity was high. Patients with dementia and static lesions performed more cognitive training. Face-to-face sessions were associated with higher intensities. The combination of classical methods with information technology systems seems to ensure greater training intensity.

## Introduction

Cognitive deficits are a common expression of highly prevalent neurological and psychiatric conditions that may affect individuals of all ages and usually have a long-lasting course [1]. This group of diseases includes Alzheimer’s and vascular dementias, stroke, Parkinson’s disease, traumatic brain injury, multiple sclerosis, bipolar disease, schizophrenia, attention deficit hyperactivity disorder, and all sorts of developmental delays [1-4].

Health systems in general are developing more targeted approaches to these conditions, like adult memory clinics, developmental clinics, comprehensive rehabilitation centers, and community-based approaches, directed at either the older population with neurodegenerative diseases [5] or school-age children with learning disabilities [3,6]. All these strategies aim to improve care, mainly through a combination of prompt detection of cognitive deficits in populations at risk and early reference and therapeutic interventions. In spite of the huge efforts to organize and improve care, both for patients and their caregivers, most of these conditions share some ominous characteristics. They are chronic and to date have no substantial pharmacological treatments [7,8].

In this context, cognitive training has been playing an ever-increasing role in the treatment of patients with cognitive deficits. More and more studies have reported some beneficial effects of cognitive training in ageing [9], mild to moderate Alzheimer’s disease and vascular dementia [10], Parkinson’s disease [11], stroke and brain injury [12], multiple sclerosis [13,14], depression, or schizophrenia [15]. In addition, some data gathered also support the idea that improvements attributed to training may generalize beyond task-specific skills [16-18], but this remains controversial due to the lack of randomized trials with appropriate controls [10,19,20]. Mostly due to methodological issues, the evidence gathered is far from providing a clear demonstration of the benefits of cognitive training and much effort is warranted to improve the design of future interventions and trials [10,21-24].

In addition, scientific discussion in the field has been raising some additional questions: (1) how to deliver this type of treatment efficiently to larger numbers of patients in need of it, (2) how to monitor and control its effects over long periods of time in real-life clinical settings, and (3) how to accommodate the increasing knowledge of neuroplastic properties of the brain and future neuro-pharmacological tools [21,25,26].

Since the number of patients that could be eligible for this type of treatment is ever increasing, it is essential to develop and validate new strategies that may improve access without elevating the costs to deliver such care [6,27]. The incorporation of computers and information technology-based systems in our current practice may optimize cognitive interventions, namely their intensity, patient adherence, and quality of professional monitoring [28-31].

We have been working on a previously described Web-based cognitive training system, “COGWEB”, since 2005. Over the years, its characteristics were tailored to address major needs identified in a memory clinic setting [32-34]. This clinic organizes and delivers care to a population of 400,000, and is based in a hospital institution with clinical and research activities.

With the present study, we aimed to analyze aspects of the quality of the cognitive training delivered, specifically, adherence and continued use of the training program in the most important subgroups of diseases attending an ordinary memory clinic setting. This was a follow-up study, focused on the investigation of the intensity of cognitive training achieved and patient adherence to treatment, using COGWEB to deliver home-based cognitive training over long periods of time.

## Methods

### Clinical Setting and Patient Selection

The study was based in a memory clinic that provides care to neurologic and psychiatric patients of all ages (adult and pediatric) with cognitive impairment, irrespective of their baseline disease. The resident staff members include neurologists and neuropsychologists, who collaborate with other departments in a tertiary hospital. Patients are referred to this clinic by other neurologists, neurosurgeons, psychiatrists, rehabilitation medicine physicians, pediatricians, internists, or general practitioners. From this outpatient memory clinic, consecutive patients that fulfilled all of the following inclusion criteria were selected: (1) medical diagnosis of a neurologic or psychiatric condition known to produce cognitive impairment, (2) cognitive deficits confirmed by comprehensive neuropsychological evaluation using tests validated for the Portuguese population, covering domains such as attention, memory, language, executive functions, and constructional ability and selected on the basis of pathology and patient characteristics (scores were reviewed by two senior neuropsychologists and each patient was classified as having or not having a deficit in each cognitive domain), (3) at least four years of formal education completed and ability to use personal computers and information technology applications, (4) favorable opinion of the attending physician and neuropsychologist toward enrollment in cognitive training activities, (5) no sensory or physical deficiency that could prevent the independent use of personal computers and information technology applications (eg, blindness, hemiplegia, or amputation), and (6) informed consent from both the patient and relative.

There were no limits of age for inclusion. Patients were first proposed by their attending physician for enrollment in cognitive rehabilitation strategies between July and December 2011. For data analysis, only the patients that had started their treatment at least four weeks before the end of the study (18 months after study beginning) were considered. This was done to guarantee a minimum follow-up time for the within-subjects adherence analysis. During the enrollment period, 240 patients were assessed at the clinic for the first time, of which 30 were classified as not having cognitive impairment. Of those remaining, 80 did not fulfill the required level of education or ability to use personal computers. Additionally, patients were deemed ineligible due to the severity of their disease or comorbidities (n=48), sensory or physical deficiency complicating stroke, diabetes, or cataracts (n=7), and no available relative to sign the informed consent (n=3).

Due to the heterogeneity of the conditions at this memory clinic [32], and to facilitate the analysis of data, patients were grouped according to their baseline pathology into four groups: (1) neurodegenerative diseases (eg, mild stages of Alzheimer’s disease, frontotemporal dementia, or Parkinson’s disease), (2) memory complaints with depressive symptoms, (3) static brain lesions (eg, stroke, traumatic brain injury, or encephalitis), and (4) other diseases (eg, epilepsy, inflammatory diseases, schizophrenia, or attention deficit hyperactive disorder).

### Ethical Issues

All patients and caregivers understood the purpose of the study and provided written informed consent. Approvals from the referring neurologists were also obtained to guarantee that the expectations of patients and caregivers were properly managed. This study was approved by the hospital review board and ethics commission (Hospital São Sebastião, Centro Hospitalar de Entre o Douro e Vouga, Santa Maria da Feira, Portugal).

### Cognitive Intervention

#### Main Characteristics of COGWEB

The COGWEB system allows for the implementation of personalized cognitive training programs remotely, in the patient’s living environment, under continuous supervision by experienced neuropsychologists [32]. The version used for this study was composed of 27 independent exercises in a computerized game format, developed to train various degrees of cognitive defects from mild to more severe impairments. Each exercise is organized primarily around a specific cognitive function, such as attention, executive functions, memory, language, praxis, gnosis, and calculus. Exercise progression is automatic through several levels of difficulty that change in accordance with the patient’s performance and are coupled with support messages in real-time. The different degrees of difficulty are obtained through the manipulation of some features such as the number and type of items per level, their intrinsic complexity, or the interval between stimuli. All exercises use random, non-sequential stimuli to prevent memorization and maintain motivation between sessions. There are also several progress graphs (eg, right answers vs wrong answers, levels completed, global training time, or accesses) that are used to motivate patients after revision by the professional in charge [32,34].

#### Cognitive Training Design and Methods Used

The activities concerning cognitive training plans were all supervised by the resident neuropsychologist, who also conducted comprehensive neuropsychological assessments according to the patient medical diagnosis and using tests validated for the Portuguese population. All patients performed Web-based cognitive training, using the COGWEB system [32,34]. The training sessions were performed outside the hospital, predominantly at patients’ homes or other comfortable family or social settings. The neuropsychologist tailored the cognitive training plan to the patients’ medical conditions and cognitive deficits, thus contents of the training sessions varied during the course of the rehabilitation program. Sessions could include exposure to different combinations and proportions of exercises focused either on memory, executive functioning, attention, language, calculation, or constructive ability. The personalization of the cognitive training plans included the following possibilities (COGWEB system features): (1) recommended duration of each daily session, (2) number of sessions per week, (3) time of the day where most training should take place (morning or afternoon), (4) type, number, initial level of difficulty, and duration of each exercise (from a pool of 27) that composed the sessions, (5) frequency of adjustments to the exercises prescribed, and (6) frequency of progress reports from the neuropsychologist to the patient/caregiver. Patients were instructed to complete a minimum number of sessions per week (7 sessions, minimum of 30 minutes each). These could be performed at the patient and caregiver’s convenience, at any time of the day in consecutive days or up to 4 sessions per day. Anything below this limit was considered non-adherence. There were no restrictions or indications of a maximum time of treatment per week.

Based on the clinical judgment of the neuropsychologists and attending physicians, some patients had their training programs based primarily on weekly face-to-face sessions with a neuropsychologist, either individualized or group sessions with an average duration of 60 minutes. Their internal organizations were defined by the neuropsychologists, according to each patient’s baseline assessment and ongoing Web-based cognitive training activities. In the specific setting of the memory clinic where the study was based, face-to-face sessions are used primarily in the rehabilitation programs of younger patients with not only static brain lesions, which are usually more severe, but also with a higher potential for socioprofessional reintegration. Older patients with stroke and early dementia may also receive this type of treatment but mainly in group sessions.

### Study Flow

**Figure 1:**
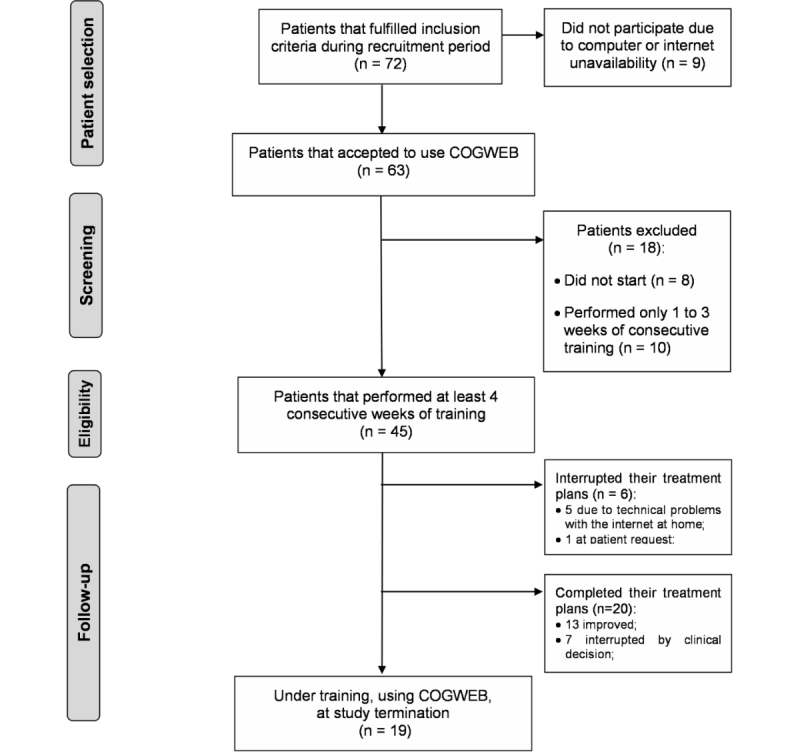
Study flowchart.

In total, 72 patients fulfilled the inclusion criteria during the recruitment period. From these, 63 patients met all conditions that allowed them to start using the COGWEB system as part of their training program. Nonetheless, 8 patients (12.7%) did not actually start and 10 (15.6%) had used the system for a period of less than four weeks at the time of the analysis (Figure 1).

The analysis was conducted on a final sample of 45 patients with a mean age of 50.7 years (SD 17.0, range 11.0-84.0), mean years of formal education of 7.8 (SD 4.9, range 4.0-17.0), and 16 (35.6%) were female. According to their baseline pathology, of the 45 patients, 9 (20.0%) had definite neurodegenerative diseases, 14 (31.1%) had memory complaints with depressive symptoms, 15 (33.3%) had static brain lesions, and 7 (15.6%) had other diseases (Table 1). Patients that interrupted their treatment plan due to technical problems with the Internet at home or by their own decision were considered as non-adherent with treatment plan (Figure 1).

The 18 patients excluded from the analysis after agreeing to use COGWEB had a mean age of 49.0 (SD 17.4, range 19.0-78.0), mean years of formal education of 10.6 (SD 4.6, range 4.0-17.0), and 42% were female. Their baseline pathologies were: 22.2% (4/18) neurodegenerative diseases, 22.2% (4/18) memory complaints with depressive symptoms, 38.9% (7/18) static brain lesions, and 16.7% (3/18) other diseases.

**Table 1:** Demographic characteristics of all groups.

Characteristics		Neuro-degenerative diseases, (n=9)	Memory complaints/ depression, (n=14)	Static brain lesions, (n=15)	Other diseases, (n=7)
Age (years), mean (SD)		61.8 (5.7)	54.8 (13.8)	44.2 (19.5)	44.6 (19.5)
Gender, n (%) male		8 (88.9)	6 (42.8)	11 (73.3)	4 (57.1)
Formal education (years), mean (SD)		6.5 (4.6)	6.1 (4.1)	9.2 (4.3)	9.9 (6.7)
**Baseline cognitive performance, n (%) with deficit**					
	Attention	9 (100.0)	12 (85.7)	13 (86.7)	4 (57.1)
	Memory	8 (88.9)	7 (50.0)	14 (93.3)	9 (100.0)
	Language	4 (44.4)	0 (0.0)	3 (20.0)	1 (14.3)
	Executive functioning	9 (100.0)	3 (21.4)	12 (80.0)	5 (71.4)
	Constructional ability	4 (44.4)	0 (0.0)	2 (13.3)	1 (14.3)
Face-to-face sessions, n (%) exposed		3 (33.3)	0 (0.0)	7 (46.7)	1 (14.3)

### Outcome Definition

The COGWEB system allowed for the continuous monitoring of the following outcomes: (1) expected time of training (minutes)—summation of the duration of all prescribed sessions of training during the follow-up period of each patient, (2) time spent training (minutes)—summation of the duration of all sessions actually performed by the patient, (3) cumulative time of training in the first and last week of follow-up (minutes/week)—time of training in the first and last weeks, (4) assiduity—difference between the minimum number of sessions prescribed and the number of sessions actually performed, expressed as the proportion of complete weeks, and (5) follow-up period (weeks)—duration of consecutive time in training for each patient, with interruptions of more than one week duration being considered as study termination and the end of the follow-up period for a particular patient. This was further categorized as withdrawal due to non-adherence or termination according to treatment plan. The first two outcomes were used to measure the intensity of cognitive training obtained and the last three to measure motivation and adherence to treatment. Cognitive training plans were also classified as exclusively Web-based if all treatment activities occurred through the COGWEB, or combined when there was weekly face-to-face cognitive training work complemented with Web-based cognitive training activities.

### Statistical Analysis

The SPSS Statistics version 21.0.0 software was used [35]. In order to characterize the global sample, mean values and standard deviations were used to describe outcomes, and parametric tests for statistical analysis were: ANOVA (analysis of variance), Student’s *t* test for independent groups, and paired *t* test for within-subject comparison of cumulative time of training in the first and last week. For subgroup description, the median and interquartile ranges (IQR) were used as they are more suitable to the size and type of distribution within each group sample. To analyze the differences in outcomes between subgroups, the Kruskal-Wallis independent samples median test was used, adjusting for multiple comparisons. The related samples Wilcoxon signed-rank test was used to compare the first and the last weeks of training within each subgroup. The independent samples Mann-Whitney *U* test was used to compare the main demographic characteristics and the outcome differences between the group with exclusive Web-based training and the group with face-to-face sessions complemented with Web-based training. Fisher’s exact test and chi-square were used to compare baseline characteristics such as gender, distribution of groups of diseases, and cognitive domains impaired, between subgroups. The effect of face-to-face sessions within subgroups of diseases was not analyzed due to the reduced sample size. Finally, an analysis of the probability to comply with the Web-based cognitive training was conducted using the Kaplan-Meier survival method in order to model the duration time of the treatment up to its interruption. Patients completing the treatment plan or undergoing training at the time of the follow-up were censored.

## Results

### Intensity of Treatment Obtained

For the duration of the entire follow-up period, patients performed on average 363.5 minutes/week (SD 136.6, range 84.7-652.6) of cognitive training activities through the COGWEB system. This was 1.7 times higher than the minimum requirement.

**Figure 2:**
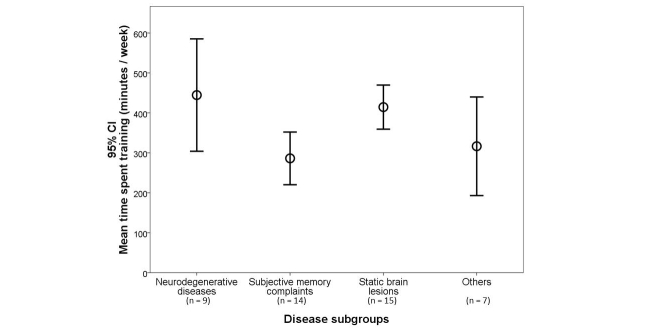
Time spent training (average in minutes/week) per disease group.

The analysis of the mean time training per week between groups of diseases revealed significant differences (Figure 2 and Table 2), with neurodegenerative diseases and static brain lesions dedicating more time to training (*H*_3_=11.41, *P*=.01). There was no association of mean time training per week with potential confounders like age (*F*_1,41_=0.86, *P*=.36), gender (*t*_42_=−1.64, *P*=.11) or education (*F*_1,41_= 0.70, *P*=.41).

**Table 2:** Indicators of intensity and adherence to treatment per major group of diseases.

	Neurodegenerative diseases, median (interquartile range)	Memory complaints/ depression, median (interquartile range)	Static brain lesions, median (interquartile range)	Other diseases, median (interquartile range)
Follow-up duration (weeks)	26 (7.8-29.8)	22.5 (7.8-33.5)	8 (5.0-25.0)	11 (7.0-18.0)
Time training per week (minutes)	479.0 (257.6-567.7)	295.3 (187.3-404.0)	423.6 (362-458)	295.6 (203.7-366.9)
Time training, first week (minutes)	555.9 (159.0-806.0)	308.3 (143.2-579.8)	501.9 (442.9-656.3)	173.3 (99.7-491.8)
Time training, last week (minutes)	394.6 (201.0-639.4)	282.5 (73.3-576.2)	376.0 (279.8-804.8)	379.5 (254.3-443.2)
Assiduity (proportion of complete weeks)	0.89 (0.53-0.96)	0.73 (0.55-0.84)	0.80 (0.75-1.0)	0.63 (0.53-0.83)

### Adherence to Treatment

The average duration of continuous cognitive training was 18.8 weeks (SD 18.9, range 4.0-55.0), and there were no statistically significant differences among groups (*H*_3_=3.40, *P*=.33) (Table 2). During the first week, the average time training was 428.7 minutes (SD 264.8, range 21.0-891.0). In the final week, this value was 414.5 minutes (SD 268.1, range 21.1-969.0). These values were not statistically different (*t*_43_=0.27, *P*=.79). There were no differences of mean time training between first and last week attributable to any of the major group of diseases (*Z*=22.00, *P*=.58 for neurodegenerative diseases; *Z=*53.00, *P*=.98 for memory complaints with depression; *Z=*63.00, *P*=.87 for static brain lesions; *Z*=14.00, *P*=1.00 for other diseases) (Table 2).

The average proportion of complete weeks of training (measure of assiduity) was 0.75 (SD 0.22, range 0.18-1.0) and there were no difference between groups (*H*_3_=4.04, *P*=.26) (Table 2).

**Figure 3:**
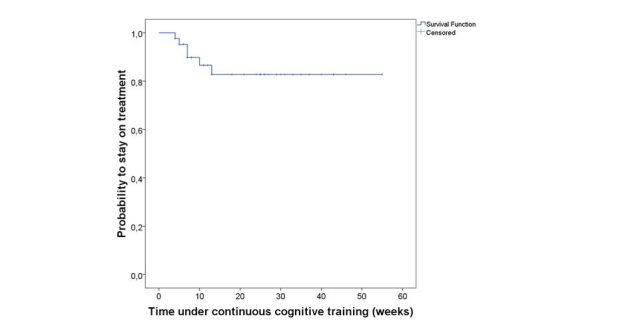
Probability of continuing with treatment over time (Kaplan-Meier survival function) for the first 60 weeks. There were no treatment interruptions after this period. Patients completing the treatment plan or undergoing training at time of follow-up were censored.

The application of the Kaplan-Meier method estimated an average duration of continuous Web-based cognitive treatment of 46.9 weeks (SD 3.03), with 95% confidence intervals of 41.3 and 52.8 weeks. At 6-month follow-up (24 weeks), 82.8% of patients complied with their treatment plan (Figure 3).

### Impact of Face-to-Face Sessions

During the follow-up period, 11/45 patients (24.4%) received weekly face-to-face sessions complemented with Web-based training (63.6%, 7/11 static brain lesions, 27.3%, 3/11 neurodegenerative, and 9.1%, 1/11 other diseases). Patients with memory complaints and depressive symptoms were excluded from this analysis since none in this subgroup was exposed to face-to-face sessions (Table 1). The baseline characteristics of the two groups are depicted in Table 3. There were no significant differences regarding age (*U*_28_=123.0, *P*=.425), formal education (*U*_28_=286.5, *P*=.718), gender (χ^2^_1_=0.6, *P*=.42), and distribution of the groups of diseases (χ^2^_2_=1.8, *P*=.42) between the two groups. The distribution of cognitive impairment by domain was also similar (Table 3).

**Table 3:** Demographic characteristics of the groups used for analysis of the impact of face-to-face sessions.

Characteristics		Exclusively Web-based training (n=20)	Face-to-face sessions complemented with Web-based training (n=11)
Age (years), mean (SD)		50.0 (19.9)	47.2 (15.6)
Gender, n (%) male		13 (65.0)	9 (81.8)
Formal education (years), mean (SD)		8.5 (5.2)	8.9 (5.1)
**Major groups of diseases, n (%)**			
	ND^a^	6 (30.0)	3 (27.3)
	SBL^b^	8 (40.0)	7 (63.6)
	OD^c^	6 (30.0)	1 (9.1)
**Baseline cognitive performance, n (%) with deficit**			
	Attention	17 (85.0)	9 (81.8)
	Memory	18 (90.0)	11 (100.0)
	Language	5 (25.0)	3 (27.3)
	Executive functioning	16 (80.0)	10 (90.9)
	Constructional ability	4 (20.0)	3 (27.3)

^a^ND: neurodegenerative diseases

^b^SBL: static brain lesions

^c^OD: other diseases

The median duration of the follow-up was higher in the group with face-to-face sessions: 26.0 weeks (IQR=7.0−43.0; min. 4.0, max. 55.0) vs 11.0 weeks (IQR=6.0−18.0; min. 4.0, max. 40.0) in the group with exclusively Web-based training. However, there was no statistical significance (*U*_28_=70.5, *P*=.145) (Table 4). The overall median time training per week in the group with face-to-face sessions was 481.4 minutes (IQR=398.4−577.3; min. 180.4, max. 652.6), while in the group with exclusively Web-based sessions it was 366.9 minutes (IQR=281.3−452.5; min. 191.3, max. 583.0). This difference had no statistical significance (*U*_28_=62.0, *P*=.07). In the last week of the cognitive intervention, significant differences were verified in the median time training between the two groups with 652.6 minutes (IQR=379.5−817.4; min. 279.8, max. 969.0) when there were face-to-face sessions vs 354.9 minutes (IQR=138.5–577.3; min. 21.1, max. 857.0) when exclusively Web-based (*U*_28_= 53.0, *P*=.027). These differences were not present in the first week of training (*U*_28_=106.0, *P*=.949) (Table 4). The overall assiduity was not different between these two groups during the study (*U*_28_=82.0, *P*=.33).

**Table 4:** Indicators of intensity and adherence to treatment per major type of treatment strategy.

	Exclusively Web-based training (n=20), median (interquartile range)	Face-to-face sessions complemented with Web-based training (n=11), median (interquartile range)
Follow-up duration (weeks)	11.0 (6.0-18.0)	26.0 (7.0-43.0)
Time training per week (minutes)	366.9 (281.3-452.5)	481.4 (398.4-577.3)
Time training, first week (minutes)	489.3 (145.9-662.9)	490.7 (173.3-655.2)
Time training, last week (minutes)	354.9 (138.5-577.3)	652.6 (379.5-817.4)
Assiduity (proportion of complete weeks)	0.75 (0.3-1.0)	0.83 (0.4-1.0)

## Discussion

### Principal Findings

This study provided data on the characteristics of cognitive training treatments using a Web-based approach in an ordinary memory clinic setting. The overall intensities of training obtained were very high, averaging 6 hours per week and exceeding 1.7 times of what was set as minimum. Furthermore, the characteristics of the system used (COGWEB) permitted uninterrupted training activities over long periods of time, with 82.8% of patients complying with treatment at 6 months. The combination of high intensity and long duration of treatment is very important to stimulate neuroplasticity in the brain [21], more so, if we consider the design of future randomized clinical trials to assess the impact of cognitive training on functional outcomes in several important diseases [21,23,36].

Significant differences were found in the mean intensity of treatment obtained between groups, with neurodegenerative diseases and static brain injury performing around 7 hours of training per week, while people with memory complaints and depressive symptoms trained close to 5 hours per week. It is important to point out that all groups performed above the minimum requirements of 30 minutes of training per day (same for all). Engaging psychiatric or neurologic patients in training or interesting leisure activities is very difficult [37]. As an example of the current state of the art, even in inpatient mental health services of developed countries, the level of activities, other than sleep, eating, or watching TV, is less than 17 minutes per day [37]. This is in high contrast with what was obtained in this study for the several groups of diseases analyzed.

During the follow-up period of the 45 patients included, and specifically comparing the first and the last week of training, the intensity of treatment did not decay and there were no important effects attributable to the major disease groups. Furthermore, follow-up duration between major groups of diseases did not differ. Although neurodegenerative disease patients had a tendency to have longer follow-up periods (around 7 months), this could be explained only by clinical reasons, with static brain lesions being prescribed shorter periods of training. These latter findings may be due to the reduced sample size for subgroup analysis.

An interesting finding of this study was the effect of weekly face-to-face sessions on the overall intensities of Web-based cognitive training activities. The group exposed to face-to-face sessions performed, on average, 2 additional hours of training per week during the entire duration of the follow-up period. This difference was not present in the first week of training, but was built over time and achieved a value of 4 hours and statistical significance in the last week of training. There was a trend for longer follow-up periods in the group with face-to-face sessions, but not achieving statistical significance. These findings are in accordance with some critical analysis of the impact of computerized cognitive training activities and the need to prevent excessive isolation of patients during treatment [38-42]. In future studies, if the intensity of treatment and adherence are to be maximized, the inclusion of some kind of periodic face-to-face individual or group session is warranted. Nonetheless, to clarify the impact of different methods of face-to-face sessions (eg, individual, group, weekly, monthly) and whether they are reproducible between groups of diseases, further studies are necessary.

### Limitations

The limitations of this study are mainly inherent to the uncontrolled nature and single center design, which impose some restrictions on the generalizability of the findings. In this respect, it is important to note that from the 240 patients initially assessed, 80 (33.3%) did not fulfil the required levels of literacy or ability to use personal computers and information technology applications. Furthermore, among the patients that fulfilled inclusion criteria, 9 out of 72 did not participate due to personal computer or Internet unavailability and 8 out of 63 did not start after agreeing to participate. These values may reflect the low literacy levels and barriers in patient access to information technology at home, in this segment of the Portuguese population [43]. Although the trends are changing [44], these aspects are still significant in the population aged over 50 and must be taken into consideration in the implementation of this type of cognitive intervention in clinical practice or future research.

In addition, the focus of this work was on obtaining data on the intensity and adherence to treatment and for that reason blinded information on cognitive baseline or outcome measures was not collected. The patient’s diagnosis only conveys indirect information on patient deficits and level of impairment, with baseline cognitive performance data provided only partially addressing this limitation. Despite the inclusion criteria defined, the enrollment of patients in the study was based upon a referral by their attending physician and neuropsychologist’s judgment. They decided whether the patient would comply with treatment and also if the deficits and background literacy or cognitive reserve were suitable. Face-to-face sessions were also decided on clinical indication and not randomized. The role of the professionals in patient selection in both these situations may have biased the results in a direction consistent with the findings. Furthermore, differences between first and last week intensities may also be due to selection biases attributable to the professional intervention. The heterogeneity of diagnoses was also a potential weakness and should not be maintained in trials evaluating clinical efficacy.

Future studies must analyze the impact of up to 7 hours of cognitive training per week on global motor activities, sedentarism indexes [45], and also possible negative mental effects of uncontrolled cognitive training activities [46]. These latter aspects are similar to the risks associated with unsupervised “of-the-shelf” home rehabilitation activities and learned non-use models during aphasia or motor rehabilitation after stroke [47-49]. They may only be avoided through control of several aspects of training like activities preformed, cumulative dose of training in each cognitive domain, and specific cognitive outcomes along time.

### Conclusions

Overall, the training intensity achieved per week was high. The groups of patients with dementia and static lesions performed more cognitive training. Patients with additional face-to-face sessions achieved a higher intensity workout. The combination of classical methods with information technology-based systems like COGWEB seems to be the option that ensures greater training intensity. This method should be further explored in multicenter randomized controlled trials targeted at the most prevalent diseases like dementia, stroke, schizophrenia, or multiple sclerosis.

## Abbreviations

ANOVA: analysis of variance
COGWEB: cognitive Web-based training system
IQR: interquartile range
